# The Effect and Optimal Dosage of Dexmedetomidine Plus Sufentanil for Postoperative Analgesia in Elderly Patients With Postoperative Delirium and Early Postoperative Cognitive Dysfunction: A Single-Center, Prospective, Randomized, Double-Blind, Controlled Trial

**DOI:** 10.3389/fnins.2020.549516

**Published:** 2020-10-23

**Authors:** Wenshuai Zhao, Yanan Hu, Hui Chen, Xifan Wang, Liping Wang, Yu Wang, Xiaohong Wu, Fei Han

**Affiliations:** ^1^Department of Anesthesiology, The Third Affiliated Hospital, Harbin Medical University, Harbin, China; ^2^Department of Anesthesiology, Sun Yat-sen Memorial Hospital, Sun Yat-sen University, Guangzhou, China; ^3^Department of Anesthesiology, Heilongjiang Provincial Corps Hospital, Chinese People’s Armed Police Forces, Harbin, China

**Keywords:** dexmedetomidine, patient-controlled analgesia, postoperative delirium, postoperative cognitive dysfunction, elderly patients

## Abstract

**Background:**

Postoperative delirium (POD) and postoperative cognitive dysfunction (POCD) are common complications after major surgery among elderly patients. Dexmedetomidine (DEX) is less frequently explored for its effects in patients with postoperative neurocognitive disorders. This study investigated the effect and optimal dosage of DEX for patient-controlled analgesia (PCA) on POD and early POCD after major surgery among elderly patients.

**Methods:**

Patients in four groups received continuous infusion of DEX 0, 100, 200, and 400 μg with sufentanil 150 μg for PCA immediately after surgery. POD and POCD were assessed on postoperative days 1, 2, 3, and 7 by using the Confusion Assessment Method (CAM) and Mini-Mental State Examination (MMSE) scales. Furthermore, the incidence of POD and POCD of all the four groups in postoperative 7 days classified by high risk factors (age, education, surgical site, and surgical category), sedation level, postoperative pain intensity, and side effects were assessed.

**Results:**

The overall incidence rates of POD and early POCD 7 days after surgery were lower in the DEX 200 μg 400 μg groups than in the DEX 0 μg and 100 μg groups (*P* < 0.05). Compared with DEX 200 μg, DEX 400 μg reduced early POCD in patients who underwent open surgery (*P* < 0.05). There were no intergroup differences in the postoperative sedation level, pain intensity, and side effects.

**Conclusion:**

The continuous infusion of DEX 200 μg or DEX 400 μg in PCA significantly decreased the incidence of POD and early POCD after major surgery without increasing any side effects. Compared with DEX 200 μg, DEX 400 μg was preferred for reducing early POCD in patients who underwent open surgery.

## Introduction

Perioperative neurocognitive disorders, recognized in one form or another for more than 100 years, affect patients, particularly elderly patients, after anesthesia and surgery (Evered et al., 2018a,b). Postoperative delirium (POD), a severe psycho-organic disorder characterized by the acute onset of confusion, inattention, and a change in the level of consciousness, frequently develops in 11–51% of elderly patients who have undergone major surgery (Ely et al., 2004; Inouye et al., 2014). Delirium is associated with an increased incidence of early postoperative cognitive dysfunction (POCD), which is reported to occur in 20–50% of patients in the first postoperative week and in 10% of patients 3 months after surgery (Rasmussen, 2006; Rudolph et al., 2008). Multimodal strategies have been used to counter POD and POCD resulting from diverse causes, such as neurotransmitter imbalance, inflammation, pain, infection, metabolic abnormalities, and sleep disorders (Moller et al., 1998; Ancelin et al., 2001; Monk et al., 2008; Skvarc et al., 2018). With regard to the various pharmacological therapies for postoperative cognitive impairment, sedative hypnotics, such as benzodiazepines, melatonin, and haloperidol, have been reported to show therapeutic effects that helped reduce the incidence and duration of POD and POCD by alleviating discomfort, anxiety, and stress.

Dexmedetomidine (DEX) is a highly selective α_2_ adrenoreceptor agonist that preserves neurologic function and attenuates neuronal injury (Bell et al., 2014). It exerts a protective effect against cognitive disabilities by inhibiting the hippocampal inflammatory response and neuronal apoptosis induced by surgical trauma (Bekker et al., 2013; Qian et al., 2015). DEX sedation in the intensive care unit has been shown to have beneficial effects on sleep quality and minimal effects on respiration. It was demonstrated that scheduled intravenous administration of DEX postoperatively reduced in-hospital delirium (Subramaniam et al., 2019). Furthermore, nocturnal administration of low-dose DEX in critically ill patients reduced the incidence and duration of delirium (Skrobik et al., 2018). DEX used for patient-controlled analgesia (PCA) reduced pain and adverse effects and improved patient satisfaction after surgery (Xin et al., 2017). However, few studies have evaluated the efficiency of DEX used for PCA against POD and early POCD. Therefore, this study was designed with an aim to investigate the effects and optimal dosage of DEX for PCA to prevent POD and early POCD after major surgery in elderly patients.

## Methods

### Trial Design

This was a single-center, prospective, randomized, double-blind, controlled study conducted from 13 April 2017 to 15 December 2017, in the Department of Anesthesiology, the Third Affiliated Hospital, Harbin Medical University. The study procedures were approved by the Ethics Committee of the Third Affiliated Hospital, Harbin Medical University (KY2017-25). Written informed consent was obtained from all subjects before surgery, and the competence of the patients was established based on his/her accurate orientation to time, place, persons, and understanding of the recruiter’s description of the trial. The trial was registered with the Chinese Clinical Trial Registry (ChiCTR-IPD-17010950) prior to patient enrollment.

### Participants

Patients aged higher than 65 years who were scheduled to undergo non-cardiac major surgery (thoracic, general, genitourinary, gynecologic, and orthopedic surgeries) and whose physical status was classified as I-III based on the American Society of Anesthesiologists (ASA) system were included in this study. The exclusion criteria were as follows: regular use of opioids, sedatives, antidepressants, or anxiolytic drugs prior to the surgery; drug addiction; severe visual or hearing disorders; preoperative history of schizophrenia, epilepsy, Parkinsonism, or myasthenia gravis; inability to communicate during the preoperative period (owing to coma, profound dementia, or a language barrier); brain injury or a history of neurosurgery; serious hepatic dysfunction (Child-Pugh class C); serious renal dysfunction (undergoing dialysis before surgery); a preoperative left ventricular ejection fraction less than 50%; sick sinus syndrome, severe sinus bradycardia (<50/min), or a second-degree or greater atrioventricular block without a pacemaker; allergy to sufentanil or DEX; and a preoperative Mini-Mental State Examination (MMSE) scores of less than 17 in illiterate (uneducated) patients, less than 20 for patients with elementary education (education of ≤6 years), and less than 24 for patients with secondary education or higher (education of >6 years) (Chua et al., 2019).

### Randomization and Masking

Eligible patients were randomly allocated into the following four groups at a 1:1:1:1 ratio: DEX 0 μg group (0 μg DEX + 150 μg sufentanil + 300 ml of 0.9% saline in the PCA pump), DEX 100 μg group (100 μg DEX + 150 μg sufentanil + 300 ml of 0.9% saline in the PCA pump), DEX 200 μg group (200 μg DEX + 150 μg sufentanil + 300 ml of 0.9% saline in the PCA pump), and DEX 400 μg group (400 μg DEX + 150 μg sufentanil + 300 ml of 0.9% saline in the PCA pump). Randomization was performed using a random number table by a physician.

The physician who kept the randomization code was the only person who was unblinded to this study. The participants, care providers, and physicians who completed the preoperative and postoperative assessments were blinded to the allocation assignment throughout the study period until a follow-up examination was completed for the final analysis. In case of an emergency (unexpected or rapid deterioration in the patient’s clinical status), physicians could request unmasking of the treatment allocation to adjust or interrupt the study if necessary. The physician who completed the preoperative assessment of POD, POCD, and side effects was responsible for educating patients on the use of PCA.

### Anesthesia and Analgesia Management

The patients in all study groups were under general anesthesia. Upon arrival at the operating room, patients were monitored by performing electrocardiography, blood pressure measurements, pulse oximetry, and body temperature tests. According to the study protocol, a loading dosage of DEX (1 μg/kg) was intravenously infused over 10 min to avoid severe hypertension and bradycardia before anesthesia induction in the DEX 100 μg, DEX 200 μg, DEX 400 μg groups. The rate of DEX administration should be reduced or discontinued if any severe side effects happened according to protocol. Atropine, 0.5 mg, was given if bradycardia (defined as heart rate < 50/min) occurred. Urapidil, 12.5–25 mg was given if hypertension (defined as mean artery pressure decreased over 30% of baseline) occurred. Ephedrine, 5–10 mg, was given if bradycardia accompanied with hypotension. No detailed data were recorded on severe hypertension and bradycardia during a loading dosage of DEX (1 μg/kg) infusion in this study. General anesthesia was induced with propofol 1.0–1.5 mg/kg, sufentanil 0.3 μg/kg, and cisatracurium 0.15 mg/kg. Endotracheal intubation was facilitated after 3 min of cisatracurium administration and was connected to the ventilator. The tidal volume (8–10 ml/kg) was adjusted to maintain the end-tidal CO_2_ (EtCO_2_) level at 35–45 mmHg. The ventilation rate was 12/min. The depth of anesthesia was adjusted according to the hemodynamic response and bispectral index (BIS). Propofol 4–6 mg/kg/h and remifentanil 6–12 μg/kg/h were administered and adjusted to maintain hemodynamic stable (±30% of baseline mean artery pressure) and BIS between 40 and 60 during general anesthesia. Ephedrine, 5–10 mg, was given if needed. The neuromuscular blockade was maintained by intermittent injection of 0.05 mg/kg cisatracurium as needed. All patients in the four groups received PCA after surgery. The PCA protocol comprised continuous infusion of 4 ml/h, a bolus dose of 3 ml (if needed), and a lock-out time of 15 min in all groups. The PCA was used for up to 72 h after surgery until all of the solution had been administered in all groups. No refilling of the PCA pump in any group was allowed in the study protocol. An acute rescue analgesic drug, tramadol (100 mg i.v.), was administered when the Visual Analog Scale (VAS: 0, no pain; 10, intense pain) scores were higher than 4 after three continuous bolus infusions of the PCA.

### Outcome Evaluation

Age, sex, height, body weight, body mass index (BMI), ASA physical status, baseline vital signs, anesthesia and surgery duration, surgical site and category, fluid volume administered during surgery, blood loss, blood transfusion, and comorbidity of the patients in all groups were recorded. The primary endpoint of this study was the incidence of POD on postoperative days 1, 2, 3, and 7 in all groups. The secondary endpoints included the incidence of early POCD, sedation level, and postoperative pain intensity (determined based on the VAS score) on postoperative days 1, 2, 3, and 7 in all groups. The incidence of POD and POCD of all the four groups in postoperative 7 days was classified by high risk factors (age, education, surgical site, and surgical category). The dose of sufentanil consumed for PCA and the number of PCA attempts on postoperative day 1 were compared among the four groups. The incidence of all side effects was recorded as the safety outcome in all the groups.

The assessment of cognitive function was performed based on the Confusion Assessment Method (CAM) and MMSE scores by a trained physician who was blinded to the group assignments. The CAM scores consisted of four features: acute and fluctuating changes in mental status, inattention, disorganized or incoherent thinking, and an altered concentration of consciousness. Delirium was considered if a patient showed an acute onset of mental status changes or a fluctuating course and inattention, with either disorganized thinking or an altered level of consciousness. The MMSE consists of tests of memory (immediate and short-term), calculation, language (naming, repetition, listening and reading comprehension, and writing), visual spatial awareness, concentration, and attention. Compared with the preoperative baseline scores, postoperative MMSE scores that decreased by more than 2 points reflected POCD (Lu et al., 2017). Prior to the study, the physician was trained according to previous studies (Ely et al., 2001; Avidan et al., 2014). The physician was considered qualified when the CAM and MMSE assessments of two cognitively impaired and two non-impaired patients were in concert with those of the delirium expert. The sedation level was assessed on postoperative days 1, 2, 3, and 7 by using the Ramsay Sedation Scale (RSS: 0, not quiet, irritable; 5, deep sleep, call wake up) and the Observer’s Assessment of Alertness/Sedation Scale (OAA/S: 0, no reaction to painful stimuli; 5, ready response to name spoken in a normal tone). The dose of the acute rescue analgesic drug (tramadol) was recorded. The perioperative variables (age, education level, surgical site, and surgical category) associated with POD and POCD were assessed among the groups. The side effects of nausea, vomiting, dizziness, headache, sleepiness, bradycardia, hypotension, and respiratory depression were compared among the groups.

### Sample Size

According to previous reports, the incidence of POD is 11–50% in the first postoperative week. The incidence of POD decreased by approximately 20% when DEX was used for sedation (Liu et al., 2016). For a two-sided difference with 80% power at a 0.05 significance level (α = 0.05, β = 0.20), the minimum sample size was 70 in each group. Considering a loss-to-follow-up rate of approximately 20%, 88 participants were required in each group.

### Statistical Analysis

The data were imported into the SPSS software for analysis (version 22.0, SPSS Science, Chicago, IL, United States). Kolmogorov–Smirnov test was used to assess distribution of the variables. Homogeneity of variance was compared among the four groups by Levene tests. Quantitative variables are presented as the mean ± SD values. Level variables are presented as median values with interquartile ranges. Categorical variables are presented as percentages. The incidence of POD and POCD, sex, ASA physical status, education level, surgical site, surgical category, the number of patients with a preoperative comorbidity, and side effects among groups were analyzed using the χ^2^ or Fisher’s exact test. RSS, OAA/S, VAS, and MMSE scores were analyzed using the Kruskal–Wallis H test. The general characteristics of the patients, including age, height, weight, BMI before surgery, anesthesia duration, surgical duration, fluid infusion, blood loss, blood transfusion during the surgery, DEX and sufentanil doses consumed, and PCA attempts, were analyzed using one-way ANOVA followed by the Bonferroni test. *P* < 0.05 was considered statistically significant.

## Results

**FIGURE 1:**
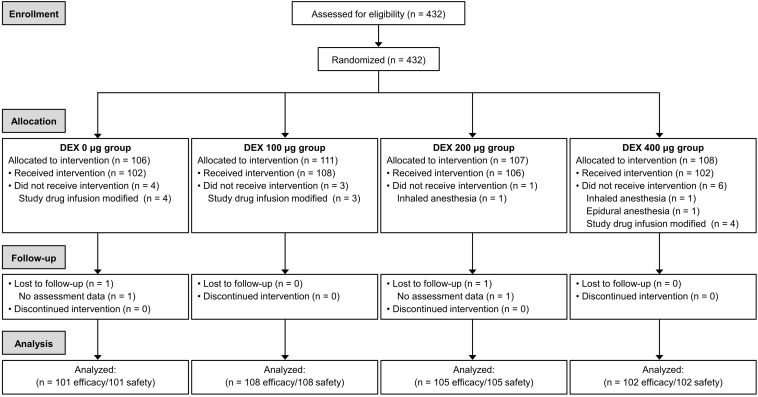
CONSOR diagram. DEX, dexmedetomidine.

In accordance with the inclusion and exclusion criteria, 432 patients were eligible and included in the study (Figure 1). However, 16 patients were subsequently excluded for analysis. Of these, 11 patients received medication violating the experimental protocol; three patients underwent different anesthetic methods; and the assessment data for two patients were not available. Thus, 416 patients were included in the analysis. The baseline characteristics of the patients (age, sex, height, body weight, BMI, ASA physical status, and preoperative comorbidity) showed no differences among all the groups (Table 1). The perioperative variables, including anesthetic duration, surgical duration, surgical site, surgical category, fluid infusion, blood loss, and blood transfusion, also showed no differences among the groups.

**TABLE 1:** Patient characteristics and perioperative features in the four groups.

	**DEX 0 μg (*n* = 101)**	**DEX 100 μg (*n* = 108)**	**DEX 200 μg(*n* = 105)**	**DEX 400 μg (*n* = 102)**	***P-*value**
Age, years	69.2 ± 4.1	70.0 ± 4.5	69.4 ± 3.9	69.3 ± 4.1	0.580
Sex, M/F	58/43	59/49	52/53	65/37	0.220
Height, cm	165.2 ± 8.0	163.8 ± 8.1	163.9 ± 7.8	166.0 ± 8.0	0.172
Body weight, kg	64.4 ± 9.3	62.6 ± 10.9	63.5 ± 10.5	64.8 ± 10.1	0.463
BMI, kg/m^2^	23.6 ± 3.3	23.3 ± 3.5	23.6 ± 3.5	23.5 ± 2.9	0.865
ASA, II/III	100/1	105/3	100/5	100/2	0.409
Anesthetic duration, h	3.5 ± 1.5	3.4 ± 1.5	3.4 ± 1.6	3.6 ± 1.5	0.937
Surgical duration, h	2.6 ± 1.4	2.6 ± 1.4	2.6 ± 1.5	2.7 ± 1.5	0.995
MMSE score before surgery	27 (24,30)	27 (24,30)	27 (24,30)	27 (24,30)	0.685
**Surgical site, *n* (%)**					0.855
Thoracic	14 (13.9%)	19 (17.6%)	18 (17.1%)	15 (14.7%)	
Abdominal	86 (85.1%)	89 (82.4%)	87 (82.9%)	87 (85.3%)	
Orthopedic	1 (0.9%)	0	0	0	
**Surgical category, *n* (%)**					0.652
Open	56 (53.5%)	66 (61.1%)	60 (57.1%)	62 (60.8%)	
MIS	45 (46.5%)	42 (38.9%)	45 (42.9%)	40 (39.2%)	
Fluid infusion, ml	1839.0 ± 843.9	1700.5 ± 537.5	1651.1 ± 590.2	1750.5 ± 568.8	0.188
Crystalloid, ml	995.5 ± 439.7	1022.7 ± 380.2	947.3 ± 444.1	996.6 ± 427.5	0.596
Colloid, ml	843.6 ± 625.1	677.8 ± 333.6	703.8 ± 413.7	753.9 ± 393.9	0.078
Blood loss, ml	126.2 ± 220.9	96.0 ± 91.9	117.5 ± 154.0	136.1 ± 136.6	0.330
**Blood transfusion**					
RBC, u	0.6 ± 1.5	0.4 ± 0.9	0.3 ± 0.7	0.5 ± 1.2	0.145
Plasma, ml	58.6 ± 146.7	33.2 ± 88.3	32.4 ± 92.5	52.7 ± 131.0	0.251
**Preoperative comorbidity, *n* (%)**					
Hypertension	17 (16.8%)	11 (10.2%)	13 (12.4%)	14 (13.7%)	0.559
Coronary heart disease	3 (3.0%)	2 (1.9%)	2 (1.9%)	3 (2.9%)	0.875
Cerebral infarction	1 (1.0%)	1 (0.9%)	3 (2.9%)	5 (4.9%)	0.242
Diabetes mellitus	8 (7.9%)	2 (1.9%)	4 (3.8%)	8 (7.8%)	0.113

*DEX, dexmedetomidine; BMI, body mass index; ASA, American Society of Anesthesiologists; MMSE, Mini-Mental State Examination; MIS, minimally invasive surgery.*

### Primary Endpoints

**FIGURE 2:**
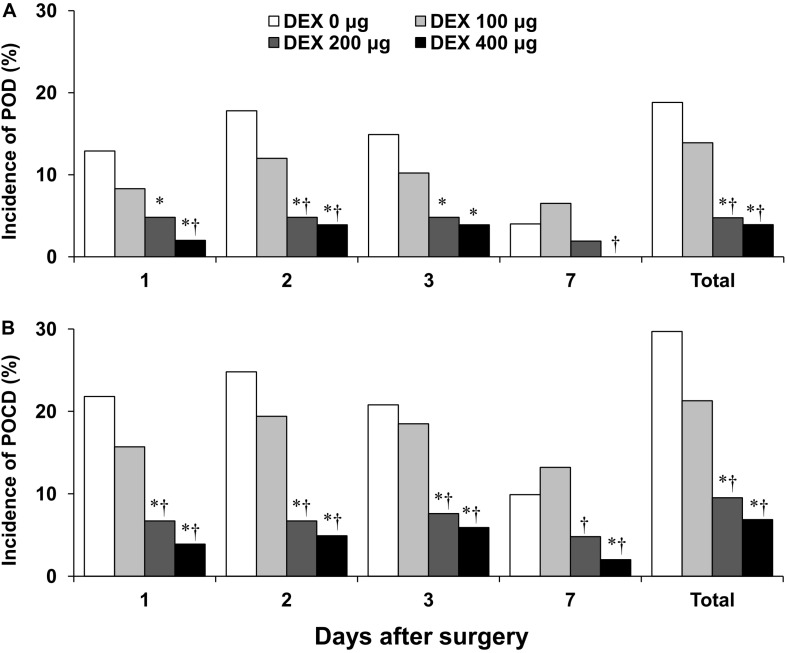
The incidence of postoperative delirium (POD) and postoperative cognitive dysfunction (POCD) in the four groups on postoperative days 1, 2, 3, and 7. **(A)** The incidence of POD. **(B)** The incidence of POCD. DEX, dexmedetomidine. ^∗^*P* < 0.05, vs. DEX 0 μg; ^†^*P* < 0.05, vs. DEX 100 μg.

The incidences of POD in the DEX 200 μg and the DEX 400 μg groups were significantly lower than those in the DEX 0 μg and DEX 100 μg groups in all 7 days after surgery (*P* < 0.05, Figure 2A). The incidences of POD in the DEX 200 μg and the DEX 400 μg groups were significantly lower than that in the DEX 0 μg group on postoperative days 1, 2, and 3 (*P* < 0.05). The incidence of POD in the DEX 200 μg group was significantly lower than that in the DEX 100 μg group on postoperative day 2 (*P* = 0.047). The incidence of POD in the DEX 400 μg group was significantly lower than that in the DEX 100 μg group on postoperative days 1, 2, and 7 (*P* < 0.05). The incidences of POD in the DEX 200 μg and the DEX 400 μg groups were significantly lower than those in the DEX 0 μg and the DEX 100 μg groups among patients aged over 70 years and in patients educated ≤ 9 years 7 days after surgery (*P* < 0.05, Table 2). The incidences of POD in the DEX 200 μg and the DEX 400 μg groups were significantly lower than that in the DEX 0 μg group among patients undergoing abdominal surgery and among those undergoing open surgery 7 days after surgery (*P* < 0.05). The incidence of POD in the DEX 400 μg group was significantly lower than that in the DEX 100 μg group for patients undergoing open surgery within 7 days after surgery (*P* = 0.02). The subtypes of POD, including hyperactivity, activity inhibition, and mixed type, showed no significant differences in all groups. There was no other difference in the incidence of POD in all groups.

**TABLE 2:** The incidence of POD and POCD of all the four groups in postoperative 7 days classified by high risk factors.

	**DEX 0 μg (*n* = 101)**	**DEX 100 μg (*n* = 108)**	**DEX 200 μg (*n* = 105)**	**DEX 400 μg (*n* = 102)**	***P-*value**
**POD**					
**Age, *n* (%)**					
65–70 years	10(14.5%)	5(7.4%)	3(4.1%)	3(4.1%)	0.061
>70 years	9(28.1%)	10(25.0%)	2(6.3%)^*†^	2(6.9%)^*†^	0.027
**Education, *n* (%)**					
>9 years	2(8.0%)	1(4.2%)	1(3.3%)	3(9.7%)	0.720
≤9 years	17(22.4%)	14(16.7%)	4(5.3%)^*†^	2(2.8%)^*†^	<0.001
**Surgical site, *n* (%)**					
Thoracic	3(21.4%)	3(15.8%)	1(5.6%)	0(0.0%)	0.280
Abdominal	15(17.4%)	12(13.5%)	4(4.6%)*	5(5.7%)*	0.013
**Surgical category, *n* (%)**					
Open	12(21.8%)	11(16.7%)	4(6.7%)*	2(3.2%)^*†^	0.006
MIS	7(15.6%)	4(9.5%)	1(2.2%)	3(7.5%)	0.162
**POCD**					
**Age, *n* (%)**					
65–70 years	17(24.6%)	10(14.7%)	7(9.6%)	5(6.8%)*	0.012
>70 years	13(40.6%)	13(32.5%)	3(9.4%)^*†^	2(5.9%)^*†^	0.002
**Education, *n* (%)**					
>9 years	2(8.0%)	3(12.5%)	1(3.3%)	3(9.7%)	0.653
≤9 years	28(36.8%)^#^	20(23.8%)	9(12.0%)*	4(5.6%)^*†^	<0.001
**Surgical site, *n* (%)**					
Thoracic	3(21.4%)	4(21.1%)	1(5.6%)	1(6.7%)	0.358
Abdominal	26(30.2%)	19(21.3%)	9(10.3%)*	6(6.9%)^*†^	<0.001
**Surgical category, n (%)**					
Open	16(28.6%)	15(22.7%)	9(16.7%)	3(4.8%)^*†‡^	0.004
MIS	14(31.1%)	8(19.0%)	1(2.2%)^*†^	4(10.0%)*	0.001

POD, postoperative delirium; POCD, postoperative cognitive dysfunction; DEX, dexmedetomidine; MIS, minimally invasive surgery. * *P* < 0.05, vs. DEX 0 μg. †*P* < 0.05, vs. DEX 100 μg; ‡*P* < 0.05, vs. DEX 200 μg. ^#^*P* < 0.05, vs. the patients with education > 9 years at the same DEX dosage.

### Secondary Endpoints

The incidences of early POCD in the DEX 200 μg and the DEX 400 μg groups were significantly lower than those in the DEX 0 μg and the DEX 100 μg groups within 7 days after surgery (*P* < 0.05, Figure 2B). The incidence of early POCD in the DEX 200 μg group was significantly lower than that in the DEX 0 μg group on postoperative days 1, 2, and 3 (*P* < 0.01). The incidence of early POCD in the DEX 400 μg group was significantly lower than that in the DEX 0 μg group on postoperative days 1, 2, 3, and 7 (*P* < 0.05). The incidences of early POCD in the DEX 200 μg and the DEX 400 μg groups were significantly lower than that in the DEX 100 μg group on postoperative days 1, 2, 3, and 7 (*P* < 0.05). The incidence of early POCD in the DEX 400 μg group was significantly lower than that in the DEX 0 μg group in patients aged ≤ 70 years within 7 days after surgery (*P* < 0.05, Table 2). The incidences of early POCD in the DEX 200 μg and the DEX 400 μg groups were significantly lower than those in the DEX 0 μg and the DEX 100 μg groups in patients aged > 70 years within 7 days after surgery (*P* < 0.05). The incidence of early POCD in patients educated > 9 years was significantly lower than that in those educated ≤ 9 years within 7 days after surgery (*P* = 0.01). The incidences of early POCD in the DEX 200 μg and the DEX 400 μg groups were significantly lower than those in the DEX 0 μg group in patients educated ≤ 9 years, in those undergoing abdominal surgery, and in those undergoing minimally invasive surgery (MIS) within 7 days after surgery (*P* < 0.05). The incidence of early POCD in the DEX 400 μg group was significantly lower than that in the DEX 100 μg group in patients aged over 70 years, educated ≤ 9 years, and undergoing open surgery within 7 days after surgery (*P* < 0.05). The incidence of early POCD in the DEX 200 μg group was significantly lower than that in the DEX 100 μg group in patients undergoing MIS within 7 days after surgery (*P* = 0.01). The incidence of early POCD in the DEX 400 μg group was significantly lower than that in the DEX 200 μg group in patients undergoing open surgery within 7 days after surgery (*P* = 0.04). There were no other significant differences in the incidence of early POCD among the groups. The RSS and OAA/S scores showed no significant differences among the four groups (Table 3). The VAS scores showed no significant differences among groups. The dosage of sufentanil consumed showed no significant difference in all groups on postoperative day 1. The number of PCA attempts showed no significant difference in all groups on postoperative day 1. No acute rescue analgesic drugs were needed for any of the groups.

**TABLE 3:** RSS, OAA/S, and VAS scores and the usage of PCA and subtypes of POD in the four groups on postoperative days 1, 2, 3, and 7.

	**DEX 0 μg (*n* = 101)**	**DEX 100 μg (*n* = 108)**	**DEX 200 μg (*n* = 105)**	**DEX 400 μg (*n* = 102)**	***P-*value**
**RSS**					
D 1	2 (2,2)	2 (2,2)	2 (2,2)	2 (2,2)	0.961
D 2	2 (2,2)	2 (2,2)	2 (2,2)	2 (2,2)	0.961
D 3	2 (2,2)	2 (2,2)	2 (2,2)	2 (2,2)	0.638
D 7	2 (2,2)	2 (2,2)	2 (2,2)	2 (2,2)	0.552
**OAA/S**					
D 1	5 (5,5)	5 (5,5)	5 (5,5)	5 (5,5)	0.260
D 2	5 (5,5)	5 (5,5)	5 (5,5)	5 (5,5)	0.260
D 3	5 (5,5)	5 (5,5)	5 (5,5)	5 (5,5)	0.591
D 7	5 (5,5)	5 (5,5)	5 (5,5)	5 (5,5)	0.590
**VAS**					
D 1	3 (3,3)	3 (3,4)	3 (3,4)	3 (3,4)	0.769
D 2	3 (3,3)	3 (3,3)	3 (3,4)	3 (3,3)	0.857
D 3	2 (2,3)	2 (2,3)	2 (2,3)	2 (2,3)	0.543
D 7	0 (0,1)	0 (0,1)	0 (0,1)	0 (0,1)	0.646
**PCA pump on D 1**					
Consumed DEX, μg	0	34.3 ± 4.8	67.7 ± 8.4	137.6 ± 17.9	<0.001
Sufentanil, μg	51.8 ± 7.8	51.1 ± 5.8	50.8 ± 6.3	51.6 ± 6.7	0.677
PCA attempts, *n*	2.4 ± 3.3	1.8 ± 2.6	1.8 ± 2.7	2.5 ± 3.5	0.232
**Subtypes of POD**					
Hyperactivity	10 (52.6%)	6 (40.0%)	3 (60.0%)	0 (0.0%)	0.167
Activity inhibition	8 (42.1%)	7 (46.7%)	1 (20.0%)	5 (100.0%)	0.069
Mixed type	1 (5.3%)	2 (13.3%)	1 (20.0%)	0 (0.0%)	0.572

*Data are presented as the median (interquartile range). RSS, Ramsay sedation score; OAA/S, Observer’s Assessment of Alertness/Sedation Scale; VAS, Visual Analog Scale; PCA, patient-controlled analgesia; POD, postoperative delirium; DEX, dexmedetomidine; D 1, 1 day after surgery; D 2, 2 days after surgery; D 3, 3 days after surgery; D 7, 7 days after surgery.*

### Safety Outcomes

The incidences of postoperative nausea, vomiting, dizziness, headache, sleepiness, and respiratory depression showed no significant differences among all groups on postoperative days 1, 2, 3, and 7 (Table 4). The incidences of bradycardia and hypotension during postoperative PCA showed no significant differences in the DEX groups compared to the DEX 0 μg group.

**TABLE 4:** Safety outcomes in the four groups.

	**DEX 0 μg (*n* = 101)**	**DEX 100 μg (*n* = 108)**	**DEX 200 μg (*n* = 105)**	**DEX 400 μg (*n* = 102)**	***P-*value**
Nausea, vomiting	15(14.9%)	11(10.2%)	10(9.5%)	9(8.8%)	0.539
Dizziness	5(5.0%)	1(0.9%)	2(1.9%)	4(3.9%)	0.276
Headache	1(1.0%)	1(0.9%)	2(1.9%)	0(0.0%)	0.903
Sleepiness	1(1.0%)	3(2.8%)	3(2.9%)	6(5.9%)	0.269
Bradycardia	3(3.0%)	8(7.4%)	8(7.6%)	8(7.8%)	0.395
Hypotension	6(5.9%)	15(13.9%)	6(5.7%)	7(6.9%)	0.092
Respiratory depression	0(0.0%)	0(0.0%)	0(0.0%)	0(0.0%)	–

*DEX, dexmedetomidine.*

## Discussion

Postoperative delirium and POCD are two stages of the same underlying process of decreased cognitive reserves. POCD, part of a cognitive trajectory toward long-term cognitive impairment, often follows POD (O’Brien et al., 2017). Both diseases share a mutual pathogenesis of neuroinflammation, oxidative stress, and challenging postoperative pain triggered by surgical trauma (Cibelli et al., 2010; Aydogan et al., 2013). DEX was reported to improve the ability of patients to counteract surgical stress and inflammatory reactions and subsequently delay the onset of POD and shorten the duration of POCD. Evidence exists for the neural anti-inflammatory properties of DEX, although human trials have yielded inconsistent results concerning its use for delirium and POCD (Li et al., 2015; Deiner et al., 2017; Carr et al., 2018). DEX is commonly used in PCA for postoperative analgesia and recovery. However, few studies have demonstrated the safety and efficacy of PCA-based DEX in reducing postoperative cognitive decline. This study is the first to investigate the effect and optimal dosage of DEX used for PCA to prevent POD and early POCD in elderly patients after major surgery. Our results suggest that, in comparison with DEX 0 μg and DEX 100 μg, with the same dosage of sufentanil, an infusion of DEX 200 μg or DEX 400 μg as PCA for postoperative analgesia over 3 days significantly decreased the incidences of POD and early POCD without increasing side effects in elderly patients undergoing major surgery. In comparison with DEX 200 μg, DEX 400 μg was preferred in patients undergoing open surgery by reducing early POCD. Furthermore, unlike the other groups, as a subtype of POD, hyperactive POD was not found in the DEX 400 μg group (consistent with a previous report) (Carrasco et al., 2016).

It was suggested that POD may be associated with postoperative pathological changes in the white matter, while a reduction in thalamic and hippocampal volumes contributed to POCD (Huang et al., 2018). An age-related cognitive decline often follows variations in regional brain volume, serotonin receptor binding, accumulation of neurofibrillary tangles, and concentrations of various brain metabolites (Kadota et al., 2001; Fotenos et al., 2005). DEX enhances arousal, nociception, attention, and sedation by binding complex protein receptors located in different brain regions (Langer, 2015). Attention is particularly affected during delirium and was shown to be improved by DEX through the norepinephrine pathways in the brain network using positron emission testing. In contrast to delirium, POCD is characterized by subtle deficits in one or more discrete domains and is improved by DEX by increasing the expression of PSD95 and reducing the expression of Aβ and p-Tau proteins in the hippocampus and prefrontal cortex (Zhang et al., 2018).

In this study, patients with an educational level ≤ 9 years were at a higher risk of POD and POCD. DEX 200 μg or 400 μg showed therapeutic advantages over POD and POCD in patients with an education of ≤ 9 years. A lower level of education represents lower cognitive reserves in the elderly, which constitutes the starting point of a causal chain leading to cognitive decline (Wilson et al., 2019). In comparison with the brain networks of individuals with a high cognitive reserve, those of individuals with a low cognitive reserve are thought to be associated with a poorer ability to cope with the disruptions caused by reduced efficiency and flexibility (Foubert-Samier et al., 2012). Patients with lower levels of education may be exposed to greater subclinical neuropathologies during surgery, such as a beta amyloid burden, which has been confirmed to be a contributor to POD and POCD (Evered et al., 2016).

In addition to the preoperative vulnerability of the patients, the effectiveness of agents in preventing cognitive decline was associated with the category of surgery and the surgical site (Monk and Price, 2011; Evered and Silbert, 2018). Abdominal surgery, dramatic hemodynamic changes, and extensive trauma, representatives of major surgery with a long duration, were hypothesized to increase the risk of cognitive impairment (Kapila et al., 2014). Fever, decreased food intake, hyperalgesia, and general fatigue were exacerbated, especially after abdominal surgery, which caused a postoperative cognitive decline through systemic cytokine release and neuroinflammation. DEX reduced postoperative complications and improved patient satisfaction after abdominal surgical procedures. Similarly, a significant preventive effect of prophylactic low-dose DEX on POD was reported in patients undergoing abdominal surgeries (Su et al., 2016). Evidence suggests that in comparison with MIS, open approaches quickly induce an inflammatory cascade and stress responses by causing greater tissue injury (Raygor et al., 2015). The intraoperative infusion of DEX reduced the release of plasma inflammatory mediators at an early postoperative time and exhibited a time delay between surgery and postoperative cognitive development. Deficient plasma concentrations of DEX resulting from inadequate PCA consumption may have hidden its anti-inflammatory and antifatigue effects. In this study, in comparison with DEX 200 μg, DEX 400 μg reduced early POCD in patients undergoing open surgery. A higher dosage of DEX 400 μg used after open surgery might attenuate the inflammatory response to surgery and reduce the capacity of patients to develop POCD.

Although a variety of scoring methods for detecting POCD have been used in different studies, the MMSE is a commonly accepted method to evaluate POCD (Sun et al., 2014; Fan et al., 2017; Edipoglu and Celik, 2019). MMSE, a simple cognitive impairment screening test scale, is reported for no sensitive to the subtle changes in cognitive function (Upadhyaya et al., 2010). However, it has high specificity in excluding the influence of objective factors such as emotional and psychological abnormalities (Benjamin and Lauterbach, 2018). The decrease in the MMSE was an independent predictor of cognitive impairment (Saito et al., 2018). In addition, it is simple and easy to carry out with high reliability, which is usually adopted by researchers. Therefore, we used MMSE to evaluate POCD in the current research. Furthermore, it was interesting in this study that the number of hypotension in the DEX 100 μg group was bigger than higher dosage groups, but the difference is statistically insignificant. Increasing concentrations of DEX resulted a biphasic dose–response relation for blood pressure. Human studies showed a transient increase in blood pressure after intravenous boluses of DEX, which is probably related to α_2_-mediated venoconstriction (Ebert et al., 2000). The effect of different dosages of DEX used for PCA on hemodynamics needs further studies.

There are several limitations to this study. First, the dosage of DEX used in the study was not calculated by body weight. However, the pharmacokinetics report showed that DEX was largely bound to plasma proteins, with its serum concentration unaffected by body weight or BMI. Administration of a dose of DEX without considering body weight has been commonly performed in other clinical research. Second, this study did not assess the subjective sleep quality promoted by DEX. A low-dose infusion of DEX was reported to promote sleep quality in non-mechanically ventilated elderly patients in the ICU following surgery. Administration of DEX potentially improved postoperative sleep in a dose-dependent manner, but this beneficial effect of DEX with a sedative dosage may be minimal. Third, considering the short hospital stays of the subjects, this analysis was limited to evaluating early neurocognitive disorders after surgery. Long-term functional assessments should be performed in follow-up studies. Fourth, the maximum evaluated dosage, DEX 400 μg, produced the optimum effect and a higher dosage was not evaluated in this study. Continuous infusion of DEX at a rate of 2–6 μg/h (total dose up to 432 μg for 3 days) after surgery can lead to adverse effects, such as dizziness, respiratory depression, bradykinesia, or hypotension (Feng et al., 2019). In addition, it was shown that higher dosage DEX applied for PCA could inhibit gastrointestinal peristalsis and prolong the recovery time of patients after surgery (Iirola et al., 2011). For safety reasons and according to the clinical practice in our hospital, DEX 400 μg was used as the maximum dose in this study. Whether higher dosage of DEX more than 400 μg used for PCA inducing even better effect or not still needs to be explored in future studies.

## Conclusion

This study suggests that, in comparison with DEX 0 μg and DEX 100 μg, a continuous infusion of DEX 200 μg or DEX 400 μg combined with the same dosage of sufentanil for up to 3 days as postoperative analgesia significantly decreased the incidence of POD and early POCD in the first 7 days after major surgery without increasing side effects. In comparison with DEX 200 μg, DEX 400 μg was preferred for reducing early POCD in patients undergoing open surgery.

## Data Availability Statement

The original contributions presented in the study are included in the article/supplementary material. Further inquiries can be directed to the corresponding author.

## Ethics Statement

The studies involving human participants were reviewed and approved by the Ethics Committee of the Third Affiliated Hospital, Harbin Medical University (KY2017-25). The patients/participants provided their written informed consent to participate in this study.

## Author Contributions

WSZ, YH, and FH designed this work. YH and FH wrote the manuscript. YH, HC, and XFW performed the experiments. WSZ, YH, HC, XFW, YW, XHW, and FH analyzed the data. All authors approved the present version of the manuscript and agreed to be accountable for all aspects of the work, including any questions related to the accuracy or integrity of any part of the work.

## Conflict of Interest

The authors declare that the research was conducted in the absence of any commercial or financial relationships that could be construed as a potential conflict of interest.
